# Pulmonary SARS-CoV-2 infection leads to para-infectious immune activation in the brain

**DOI:** 10.3389/fimmu.2024.1440324

**Published:** 2024-10-14

**Authors:** Cordelia Dunai, Claire Hetherington, Sarah A. Boardman, Jordan J. Clark, Parul Sharma, Krishanthi Subramaniam, Kukatharmini Tharmaratnam, Edward J. Needham, Robyn Williams, Yun Huang, Greta K. Wood, Ceryce Collie, Andrew Fower, Hannah Fox, Mark A. Ellul, Marie Held, Franklyn N. Egbe, Michael Griffiths, Tom Solomon, Gerome Breen, Anja Kipar, Jonathan Cavanagh, Sarosh R. Irani, Angela Vincent, James P. Stewart, Leonie S. Taams, David K. Menon, Benedict D. Michael

**Affiliations:** ^1^ NIHR Health Protection Research Unit in Emerging and Zoonotic Infections, Liverpool, United Kingdom; ^2^ Clinical Infection Microbiology and Immunology, Institute of Infection Ecology and Veterinary Sciences, University of Liverpool, Liverpool, United Kingdom; ^3^ Department of Infection Biology and Microbiomes, Institute of Infection, Veterinary and Ecological Sciences, University of Liverpool, Liverpool, United Kingdom; ^4^ Department of Health Data Science, Institute of Population Health, Faculty of Health and Life Sciences, University of Liverpool, Liverpool, United Kingdom; ^5^ Department of Clinical Neurosciences, University of Cambridge, Cambridge, United Kingdom; ^6^ Nuffield Department of Clinical Neurosciences, Medical Sciences Division, University of Oxford, Oxford, United Kingdom; ^7^ Centre for Cell Imaging, Faculty of Health and Life Sciences, University of Liverpool, Liverpool, United Kingdom; ^8^ Department of Neurology, The Walton Centre NHS Foundation Trust, Liverpool, United Kingdom; ^9^ Department of Social, Genetic & Developmental Psychiatry Centre, School of Mental Health & Psychological Sciences, King’s College London, London, United Kingdom; ^10^ NIHR Maudsley Biomedical Research Centre, King’s College London, London, United Kingdom; ^11^ Laboratory for Animal Model Pathology, Institute of Veterinary Pathology, Vetsuisse Faculty, University of Zurich, Zurich, Switzerland; ^12^ College of Medical, Veterinary and Life Sciences, University of Glasgow, Glasgow, United Kingdom; ^13^ Centre for Inflammation Biology and Cancer Immunology, Department of Inflammation Biology, School of Immunology & Microbial Sciences, Faculty of Life Sciences & Medicine, King’s College London, London, United Kingdom; ^14^ Division of Anaesthesia, Addenbrooke’s Hospital, Cambridge University Hospitals, Cambridge, United Kingdom

**Keywords:** virology, immunology, SARS-CoV-2, neurology, microglia

## Abstract

Neurological complications, including encephalopathy and stroke, occur in a significant proportion of COVID-19 cases but viral protein is seldom detected in the brain parenchyma. To model this situation, we developed a novel low-inoculum K18-hACE2 mouse model of SARS-CoV-2 infection during which active viral replication was consistently seen in mouse lungs but not in the brain. We found that several mediators previously associated with encephalopathy in clinical samples were upregulated in the lung, including CCL2, and IL-6. In addition, several inflammatory mediations, including CCL4, IFNγ, IL-17A, were upregulated in the brain, associated with microglial reactivity. Parallel *in vitro* experiments demonstrated that the filtered supernatant from SARS-CoV-2 virion exposed brain endothelial cells induced activation of uninfected microglia. This model successfully recreates SARS-CoV-2 virus-associated para-infectious brain inflammation which can be used to study the pathophysiology of the neurological complications and the identification of potential immune targets for treatment.

## Graphical Abstract

The low inoculum SARS-CoV-2 mouse model of para-infectious brain effects in the absence of active viral replication had increased cytokines (CCL4, IFNγ and IL-17A) and microglia reactivity (increased Iba1 expression and ramification index).

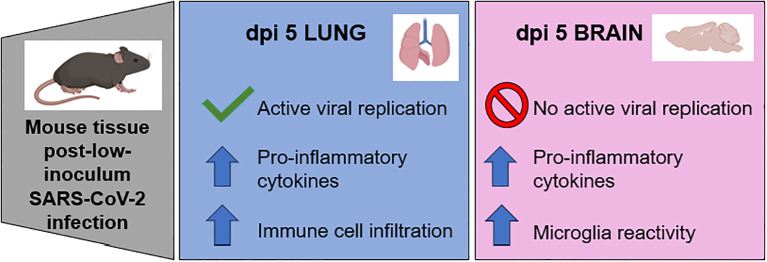

## Introduction

SARS-CoV-2 infection has been associated with a range of neurological complications. Although their incidence has decreased with widespread vaccination and effective anti-viral and anti-inflammatory treatments, they remain a significant clinical issue (1). A large retrospective study with contemporary controls found neurological complications were more common in people who had experienced COVID-19 but were also found in non-hospitalised/mild cases of COVID-19, revealing a large healthcare burden left in the wake of the pandemic (2). The different neurological complications, ranging from loss of smell to encephalitis, caused by SARS-CoV-2 infection most likely have very different aetiologies, but viral protein is seldom found in the brain parenchyma strongly suggesting that indirect effects of the virus, such as immune-mediated pathologies, are a likely potential cause (3–6). Indeed, we and others previously found that COVID-19 patients with neurological complications had elevated serum immune mediators and cytokines (IL-6, IL-12p40, IL1-RA, M-CSF, CCL2, and HGF) which correlated with serum brain injury markers (7–10).

Mouse models are important to systematically study early changes in disease and disease progression, but most models of SARS-CoV-2 have involved systemic dissemination with brain pathology that may not reflect the clinical scenarios (11–14). We established a mouse model using an inoculum of virus ten times lower than that which earlier studies have used, and looked for evidence of brain immune activation in the absence of viral replication in the brain. We also developed an *in vitro* assay to investigate how exposure of endothelial cells to viral protein can lead to cytokine-mediated indirect effects on microglia, which we hypothesize is the most common clinical scenario for how SARS-CoV-2 affects the brain (15).

## Methods

### Mouse studies of infection with SARS-CoV-2

An AWERB-approved protocol was followed for the mouse studies (University of Liverpool Animal Welfare and Ethical Review Body, UK Home Office Project Licence PP4715265). Mice were maintained under SPF barrier conditions in individually ventilated cages. Male and female 2-4 month old heterozygote hACE2-transgenic C57BL/6 mice (Charles River Laboratory) were infected intranasally with 1x10^3^ or 1x10^4^ plaque-forming units (PFU) of a human isolate of SARS-CoV-2 (Pango B lineage hCoV-2/human/Liverpool/REMRQ0001/2020) under isoflurane anaesthesia. Mice were euthanized on day 5 post infection. Brains were perfused with 30mL PBS w/1mM EDTA, then one hemisphere fixed in formaldehyde-containing PLP buffer overnight at 4°C. Brains were then subjected to a sucrose gradient– 10 and 20% sucrose for 1hr each and then 30% sucrose O/N at 4°C. Brains were then frozen in OCT by submerging moulds in a beaker of 2-methylbutane on dry ice. The other hemisphere was divided in two sagittal sections and half preserved in 4%PFA for histology and half in trizol for RNA and protein extraction. Sera was collected and frozen and then heat-inactivated at 56°C for 30 mins prior to be moved from CL3 to CL2 lab. Lung tissue was preserved in 4%PFA for histology, in trizol for RNA and protein extraction, and in PLP for cryosectioning. Cytokines were measured from tissue protein extract with Bio-rad reagents on a Bio-plex 200 following the manufacturer's protocol.

### qPCR of SARS-CoV-2 genes and mouse cytokines

Gene expression was measured from trizol isolated RNA (Invitrogen cat# 15596018, manufacturer’s protocol) using Promega’s GoTaq Probe 1-Step RT-qPCR system (cat#A6120, manufacturer’s protocol) on an Agilent AriaMx. Primers and FAM probes for SARS-CoV-2, cytokines, and housekeeping genes were purchased from IDT (**Tables 1**, **2**) with standard IDT qPCR primer/probe sets for the mouse cytokines.

**Table 1:** Primers and probes for viral genes and normalization.

Gene	Reagent	IDT Cat#	Sequence (5′–3′)
**N1**	Forward primer:	10006830	GACCCCAAAATCAGCGAAAT
	Reverse primer:	10006831	TCTGGTTACTGCCAGTTGAATCTG
	FAM probe:	10006832	ACCCCGCATTACGTTTGGTGGACC
**subgE**	Forward primer:	10006889	CGATCTCTTGTAGATCTGTTCTC
	Reverse primer:	10006891	ATATTGCAGCAGTACGCACACA
	FAM probe:	10006893	ACACTAGCCATCCTTACTGCGCTTCG
**18S**	Forward primer:	Custom	ACCTGGTTGATCCTGCCAGTAG
	Reverse primer:	Custom	AGCCATTCGCAGTTTCACTGTAC
	FAM probe:	Custom	TCAAAGATTAAGCCATGCATGTCTAAGTACGCAC

**Table 2:** Primers and probes for mouse cytokines.

Gene	IDT Primetime Cat#
IL-1RN	Mm.PT.58.43781580
IL-6	Mm.PT.58.10005566
IL-12p40	Mm.PT.58.12409997
M-CSF	Mm.PT.58.11661276
CCL2	Mm.PT.58.42151692
HGF	Mm.PT.58.9088506
CCL4	Mm.PT.58.5219433
IFNG	Mm.PT.58.41769240
IL-17A	Mm.PT.58.6531092

For mouse cytokines, the primer/probe sets listed in **Table 2** were used and the cycle was: 45°C for 15 min; 95°C for 2 min; 45 cycles of 95°C for 3 sec and 55°C for 30 sec.

The thermal cycle for N1 and the mouse cytokines was: 45°C for 15min 1x, 95°C for 2 min, then 45 cycles of 95°C for 3 secs followed by 55°C for 30 sec.

For subgenomic E: 45°C for 15 min 1x, 95°C for 2 min, then 45 cycles of 95°C for 15 secs followed by 58°C for 30 sec.

For 18S it was: 45°C for 15 min, 95°C for 2 min and 40 cycles of 95°C for 15s, 60°C for 1 min.

### Histology and confocal microscopy

Paraffin-embedded formalin-fixed tissue was sectioned to 4 um sections. Slides were baked at 60°C for 30 minutes and then stained with H&E in an autostainer. H&E slides were imaged on a Leica microscope. For immunofluorescent staining and confocal microscopy, OCT-embedded frozen tissue was sectioned to 12 um or 30 um sections (thicker sections needed for the Z-stack imaging and microglia ramification/reactivation quantification). 100% acetone was used for antigen retrieval (10 mins at room temperature). After air-drying, then PBS washing, tissue sections were permeabilized with 0.1% Triton X-100/PBS (20 mins at room temperature). After rinsing with PBS, tissue sections were blocked with Dako block (5 minutes at room temperature). After another PBS wash, primary antibodies were added at dilutions listed in table for an overnight incubation at 4°C in a humidified chamber. Tissue sections were washed twice with PBS for 5 minutes each wash. Secondary antibody (as described in **Table 3**) was added for a 2 hr room temperature incubation, followed by two 5-minute PBS washes. DAPI-mounting medium was used for coverslipping. Imaging was performed with Andor Dragonfly spinning disk confocal microscope. Marker fluorescence and microglia counts, intensity, and reactivation indices [method based on previous work (16, 17)] were quantified with Fiji (confocal microscope set up in **Table 4** and macros downloadable from the public Github repository). The reactivation index is the area of the cell divided by the projection area (the whole polygon covered by the cell). Reactivation indices of 0-1 of objects with a threshold size of 19 mm^2^ were quantified from three Z-stack images/mouse and two mice/group.

**Table 3:** Antibodies for immunofluorescent stain and confocal imaging.

Antibody target-fluorochrome	Company	Cat#	Host	Dilution Factor (for 200 µL per section)
CD45-PE	Invitrogen	12-0451-82	Rat	50
CD11b-AF647	BDBioscience	557686	Rat	200
NK1.1-AF488	BioLegend	108718	Mouse	100
CD3-FITC	Abcam	ab34722	Rat	50
GFAP	Invitrogen	41-9892-82	Mouse	100
NeuN	Merck	MAB377X	Mouse	100
CD68 unconjugated	Abcam	ab53444	Rat	200
Anti-rat AF488	Jackson Immunoresearch	712-546-153	Donkey	500
Iba1 unconjugated	Wako	019-19741	Rabbit	500
Anti-rabbit IgG-AF647	Invitrogen	A-31573	Donkey	500
Spike unconjugated	Invitrogen	703958	Human	100
Anti-human AF568	Fisher	A-21090	Goat	500

**Table 4:** Confocal microscopy settings.

Microscope component	Parameters
Microscope	Leica DMi8 with Andor Dragonfly
Light source	7-line integrated laser engine equipped with: Solid state 405 smart diode laser at 100mW: set to 10% Solid state 488 smart diode laser at 50mW: set to 35% OBIS LS 561 smart OPSS laser at 50mW: set to 2.0% OBIS LX Solid state 637 smart diode laser at 140mW: set to 7.0%
Excitation/emission optics	Dichroic mirror: Quad EM filter 405-488-561-640 Dual camera beam splitter: Dual camera dichroic 565nm long pass Emission filters: 450/50nm bandpass filter 525/50nm bandpass filter 600/50nm bandpass filter 700/75nm bandpass filter Spinning disk with 40 µm pinholes
Objective lenses	Leica objectives: 11506358 HC PL APO 40×/1.30 OIL CS2
Detector	Andor iXon Ultra 888 Ultra EMCCD Camera 1024 × 1024; 405: 500 ms exposure; 65 EM gain 488: 500 ms exposure; 65 EM gain 561: 30 ms exposure; 156 EM gain 637: 30 ms exposure; 156 EM gain Averaging: 1; Binning: 1; camera magnification 1x Nyquist Z sampling

### *In vitro* cell culture assays

The investigated cell lines were cultured according to the manufacturer’s recommendations. The mouse brain endothelial cell line, bEnd.3 (ECACC 96091929) was used between passage 22 and 29. The cells were cultured in Dulbecco’s Modified Eagle Medium (DMEM) supplemented with 2mM Glutamine, 5µM 2-Mercaptoethanol (2ME), 1mM Sodium Pyruvate (NaP), 1% Non Essential Amino Acids (NEAA), 10% Foetal Bovine Serum (FBS) and 1X penicillin/streptomycin (P/S). Primary mouse microglia (ScienCell, SC-M1900-57) were cultured on poly-L-lysine (ScienCell 0413) coated flasks in Microglia medium (ScienCell 1901) supplemented with 1% FBS, 1x microglia growth supplement (MGS, ScienCell 1952) and 1 x P/S. Primary mouse astrocytes were cultured on poly-L-lysine (ScienCell 0413) coated flasks in Astrocyte Medium (ScienCell 1801) supplemented with 2% FBS, 1x astrocyte growth supplement (AGS, ScienCell 1852) and 1 x P/S.

### *In vitro* incubation with inactivated virus and endothelial supernatants

Acid/heat inactivated SARS-CoV-2 (B.1.1.7) (NIBSC 101027) of known titre was used for all experiments. For incubation with inactivated virus, cells were seeded into either 24 well plates or 6 well plates at 125,000 cells per cm^2^ and allowed to adhere overnight. Virus dilutions were then prepared to give a ratio of 1 copy (MOI 1), 0.1 copies (MOI 0.1) or 0.01 copies (MOI 0.01) per cells plated in the culture medium. For bEnd.3 experiments, cells were incubated for 24 hours with inactivated virus, 20 µg/mL polyI:C (Merck PL530) or with untreated culture medium. For astrocyte and microglia experiments, cells were incubated for 2 hours with inactivated virus, polyI:C, untreated medium, or supernatant from bEnd.3 cells previously exposed for 24 hours to MOI 1 virus dilution. Supernatant from bEnd.3 cells was filtered with a 20 nm syringe filter directly prior to treating microglia and astrocytes to remove viral particles. After 2 hours, treatments were removed, cells were washed with 1X phosphate-buffered saline (PBS) (Capricorn Scientific CSR154) three times and then the normal culture medium was replaced. For each treatment condition (virus or supernatant) 6-7 individual well replicates were performed and for control conditions (poly:IC or untreated) 3-4 replicates were performed. After 24 hours, the supernatant from these cells was collected and cells were fixed with 4% paraformaldehyde (Sigma 16005).

### ELISA of *in vitro* supernatants

Supernatants were collected into 1 mL cryovials and stored at 4°C for no longer than 1 week prior to cytokine levels being assessed using ELISA. Kits were purchased from Invitrogen for IL-6 (88-7064), CCL2 (88-7391), IFNγ (88-7314) and performed according to manufacturer’s protocol.

### Immunostaining of *in vitro* samples

Microglia were fixed with 4% paraformaldehyde and washed 3x with 1X PBS with calcium and magnesium (PBS +/+) (Capricorn Scientific CSR1576). Cells were then incubated for 1 hour with Dakoblock (Agilent X090930-2). Cells were then washed 1x with PBS and incubated with primary antibody (Iba1 Wako Chemicals 019-19741; CD45-PE Thermo Fisher 12-0451-83) diluted in antibody diluent (1X PBS +/+, 1% BSA, 10% donkey serum (Sigma D9663), 0.1% Triton-X (Sigma X100)) overnight at 4°C. Cells were then washed 3x with 1x PBS +/+ and incubated with secondary antibody (Donkey anti-rabbit AF657, Thermo Fisher A31573) and DAPI (Thermofisher P36962) in antibody diluent and incubated in the dark for 2 hours at room temperature. Cells were then washed 3x with PBS +/+.

### *In vitro* confocal microscopy and reactivation/ramification index quantitation

Microglia were imaged at 25x magnification on a Leica DMi8 on an Andor Dragonfly spinning disk confocal microscope. 16 images per well were taken and stitched into a tilescan image using Fusion imaging software. Tilescan images were then processed in Fiji/ImageJ by thresholding the PE/647 channel and gating for individual cells (excluding cell clusters) and then analysing particles between 500-1000 µm for solidity.

### Statistical analyses

Prism software (version 9.4.1, GraphPad Software Inc.) was used for graph generation and statistical analysis. The Shapiro-Wilk normality test used to check the normality of the distribution. Data are expressed as mean ± S.E.M. The difference between two or more non-normally distributed groups was tested using Mann-Whitney U or Kruskal-Wallis tests, respectively. P ≤ 0.05 was considered statistically significant.

## Results

### Low inoculum intranasal SARS-CoV-2 infection in human ACE2 transgenic C57BL/6 mice does not cause viral replication in the brain parenchyma

**Figure 1:**
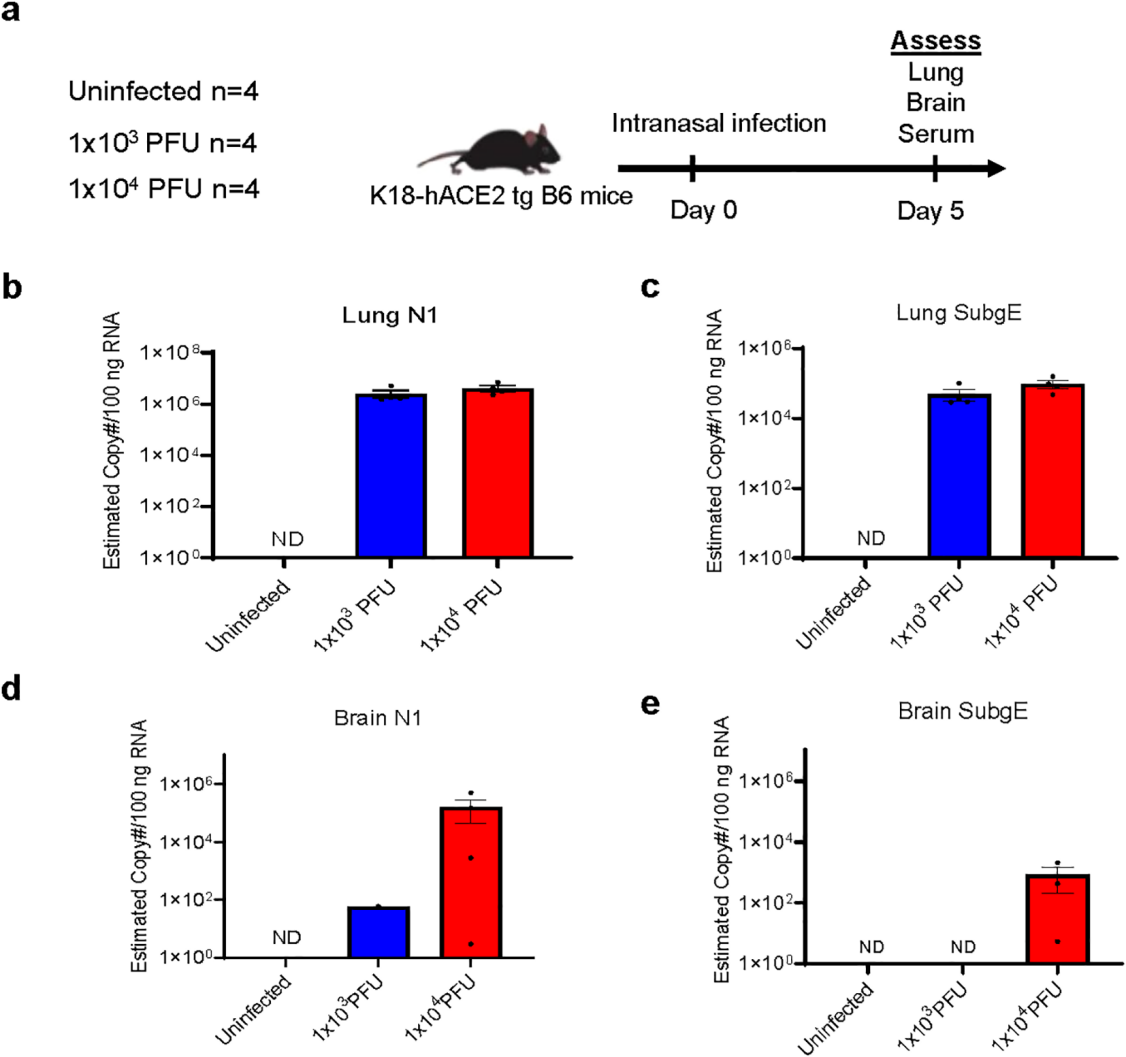
Low inoculum intranasal SARS-CoV-2 infection in human ACE2 transgenic C57BL/6 mice does not cause viral replication in the brain parenchyma. **(A)** Schema of K18 human-ACE2 transgenic C57B/6 mouse study randomised to no infection, 'low inoculum' infection at 1x10^3^ plaque-forming units (PFU) and 'high inoculum' infection at 1x10^4^ PFU, with endpoint at day 5 post-infection, showing one of two independent experiments, (n=4/group). **(B)** Real-time polymerase chain reaction (RT-QPCR) identifies SARS-CoV-2 N1 and **(C)** subgenomic E transcripts in lung homogenate confirming pulmonary infection and viral lytic replication at both inoculum of infection. **(D)** RT-QPCR of brain homogenate demonstrates that minimal SARS-CoV-2 N1 is present in the perfused brain parenchyma and there are no detectable SARS-CoV-2 subgenomic E transcripts **(E)** confirming the absence of lytic viral replication in the brain at low-dose infection. ND, not detected.

We established the mouse model by comparing intranasal infection with a low (1x10^3^ PFU) and a high (1x10^4^ PFU) inoculum of SARS-CoV-2, collecting serum, brain and lung tissues at day 5 post-infection (**Figure 1A**). At this stage, none of the mice showed weight loss (**Supplementary Figure 1A**). Both levels of infection caused pathology in the lung, with viral loads evidenced by qPCR of N1 with both low and high inocula (**Figure 1B**). There was also evidence of active viral replication in the lung, as defined by the surrogate readout of qPCR of subgenomic E which correlates with infectivity (18) (**Figure 1C**). SARS-CoV-2 N1 transcripts were detected in four out of four brains of mice that had received high inoculum of SARS-CoV-2 and in six of nine that received low inoculum (**Figure 1D**; first experiment displayed, n=4 group). However, whilst three of four high inoculum mice showed active viral transcription in the brain by the detection of subgenomic E, this was only detectable in two of nine low inoculum mouse brains (tissue from these two animals were not included in subsequent ex vivo experiments, **Figure 1E**; **Supplementary Figures 1E–G**).

H&E staining of lung and brain sections from infected mice were assessed for pathology and mononuclear cell clusters (**Supplementary Figures 1B–D**). The lungs of SARS-CoV-2 infected mice showed signs of pathology including oedema, haemorrhage, and fibrosis (**Supplementary Figure 1B**). Mononuclear cells were present with both inocula compared with uninfected mice (**Supplementary Figure 1B**. The brain tissue also had a few clusters of mononuclear cells in the frontal cortex with both inocula of SARS-CoV-2 (**Supplementary Figures 1C, D**).

### Spike protein is present in the lungs, but not brains, of 1x10^3^ PFU SARS-CoV-2 infected mice and there are differences in immune activation

**Figure 2:**
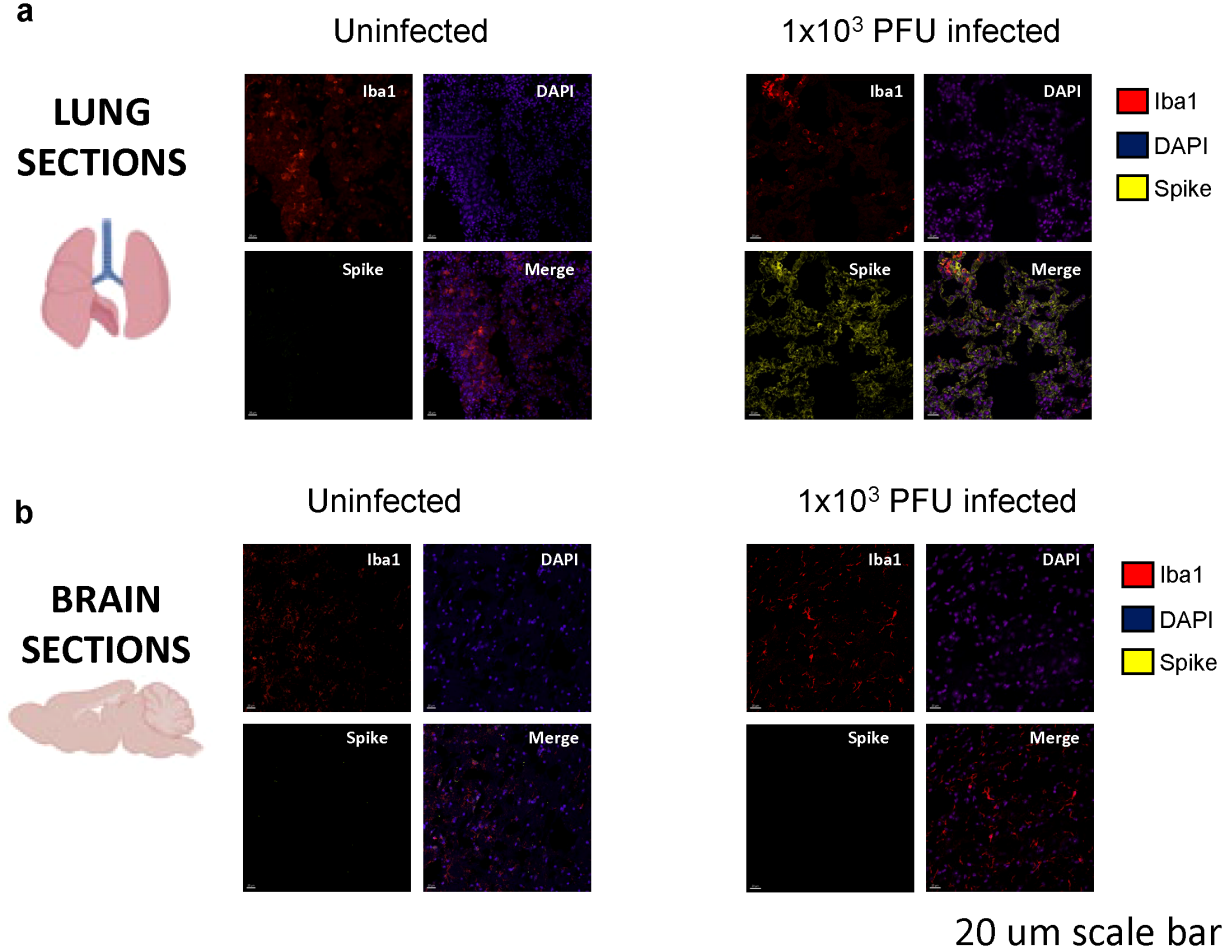
Spike protein is present in the lungs, but not in the brains of low-inoculum SARS-CoV-2 infected mice at day 5 post 1x10^3^ PFU infection. **(A)** Confocal microscopy of low-inoculum infection in this model confirms the presence of both SARS-CoV-2 spike protein (yellow) and monocyte lineage cells (red) in the lungs. **(B)** Despite the absence of spike protein (yellow) in the brain in the low-inoculum infection model, there are consistent large areas of accumulation of Iba1+ microglia (red) of perfused brain parenchyma.

**Figure 3:**
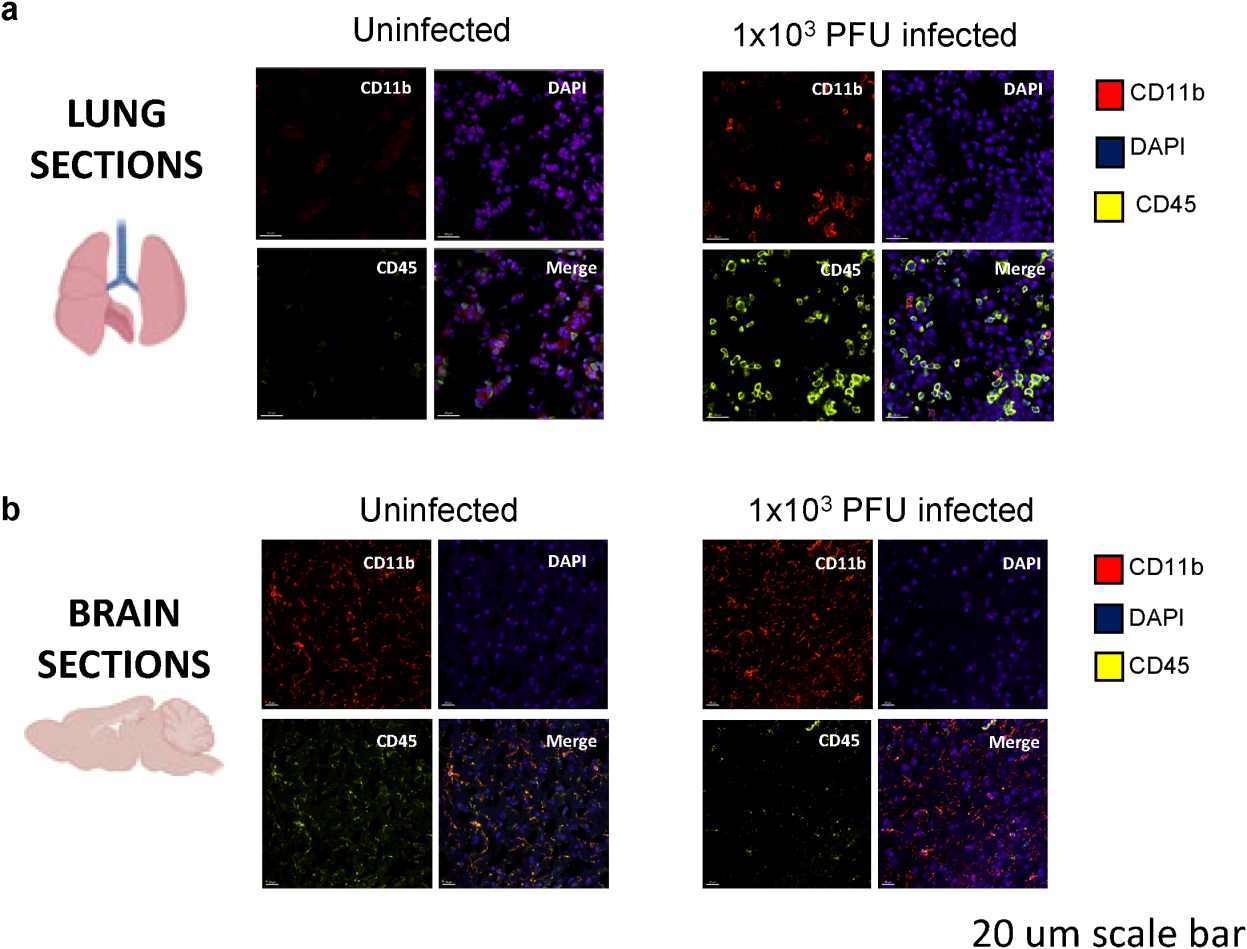
Immune activation is present in the lungs of SARS-CoV-2 infected mice at day 5 post 1x10^3^ PFU infection. **(A)** Confocal microscopy of perfused organs in the low-inoculum SARS-CoV-2 infection model identifies the presence of CD45 positive leucocytes (yellow) and occasionally CD11b positive cells of macrophage/monocyte lineage (red), shown with DAPI (blue) in the lung. **(B)** No significant differences in CD45 and CD11b expression were found when comparing uninfected and infected brains.

Immunofluorescent staining and confocal microscopy of the lungs showed large amounts of viral spike protein with increased Iba1 expression (**Figure 2A**). The lungs also showed increases in CD45 and CD11b staining compared with uninfected animals (**Figure 3A**). Confocal microscopy of ex vivo brain sections from mice, following the low inoculum, demonstrated Iba-1 staining but, in contrast to lung tissue, showed no evidence of staining for SARS-CoV-2 spike protein (**Figures 2B**, **3B**). These brains also showed no increases in numbers of CD3+ or NK1.1+ cells (**Supplementary Figures 2A, B**), or apoptotic cells (**Supplementary Figure 2C**).

### Several inflammatory mediators are elevated in the lungs and brains of 1x10^3^ PFU SARS-CoV-2 infected mice

**Figure 4:**
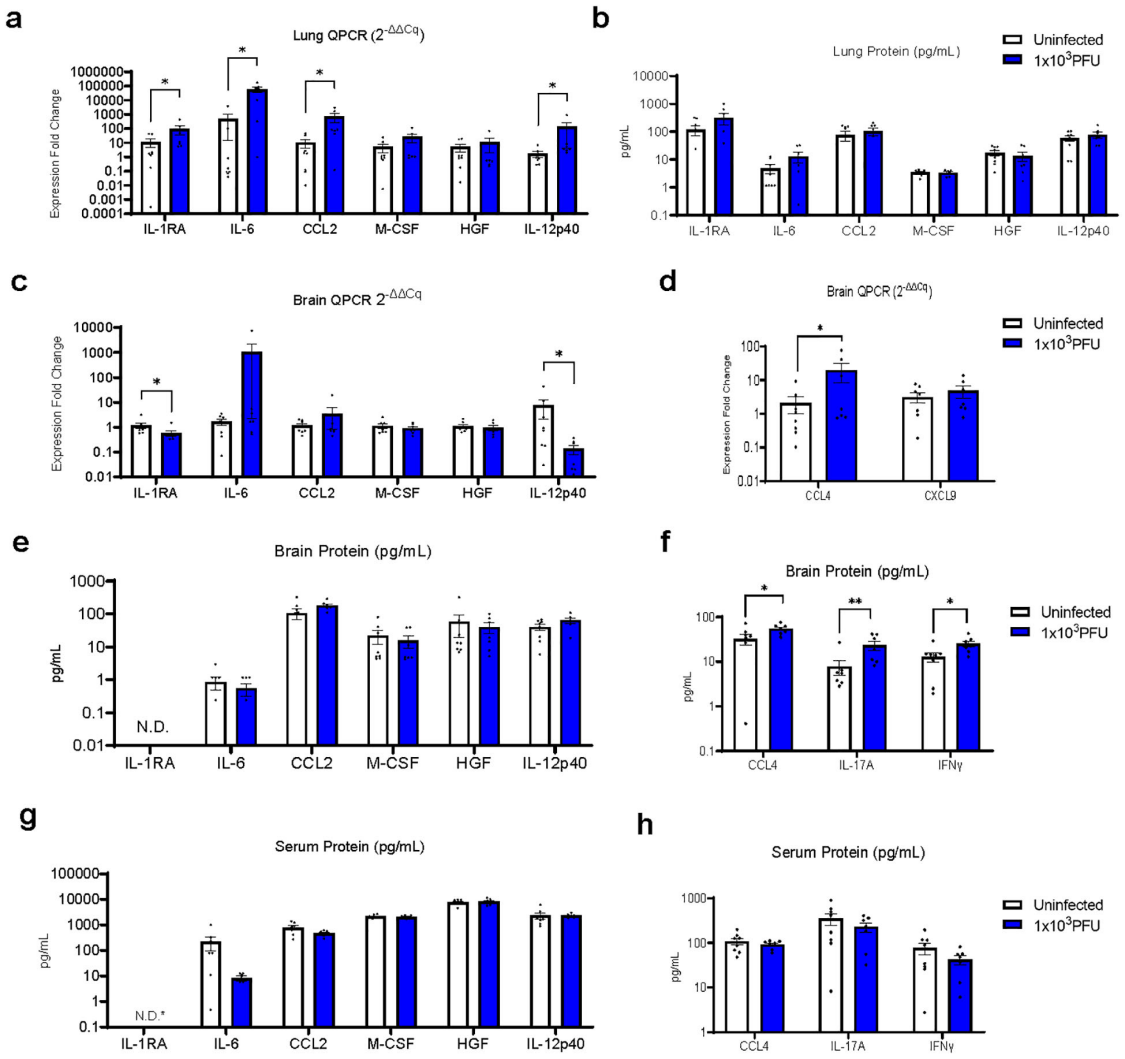
RT-QPCR of perfused lung parenchyma in the low-inoculum infection model (day 5 post 1x10^3^ PFU infection) confirms upregulation of several inflammatory mediators. **(A)** RT-QPCR of six immune mediators of interest in perfused lung. **(B)** Luminex was used to check the same six immune mediators of interest in perfused lung. **(C–F)** In this low-inoculum infection model several pro-inflammatory mediators were found to be increased in the perfused brains, including **(D)** CCL4 by RT-QPCR and **(F)** IL-17A and IFNγ by Luminex. **(G, H)** In contrast, no statistically significant differences were seen in serum cytokine protein levels. *IL-RA undetectable by ELISA in all, but one uninfected serum sample (=3.5 pg/mL). (These data are from two independent experiments combined, n=7-8/group). Pairwise comparisons by Mann-Whitney U test (*p<0.05, **p<0.01).

In order to understand the mechanisms driving this apparent para-infectious neuropathology, we assessed transcription and protein levels of inflammatory mediators in brains and lung from the seven low-inoculum infected mice that showed no evidence of local viral replication in the brain. Our previous clinical study, comparing serum immune mediators from low vs. normal Glasgow coma scale score COVID-19 patients, demonstrated that six mediators of interest were increased in serum (7). Lung tissue from the mice revealed four of these, IL1-RA, IL-6, CCL2, and IL-12p40, to be upregulated with low-inoculum infection by QPCR (**Figures 4A, B**).

The brains from low inoculum infected mice showed increased transcripts of CCL4 and decreased levels of IL-1RA and IL-12p40 (**Figures 4C, D**; **Supplementary Table 1**). Although many brain cytokine proteins were not different between uninfected and low inoculum mice, CCL4, IFNγ and IL-17A were increased (**Figures 4E, F**; **Supplementary Table 2**). Consistent with an intra-cerebral local response, there were no increases in any of these cytokines in the mouse sera (**Figures 4G, H**).

### Brains from low inoculum SARS-CoV-2 infected mice showed immune activation and increases in microglial reactivity despite the absence of active SARS-CoV-2 replication

**Figure 5:**
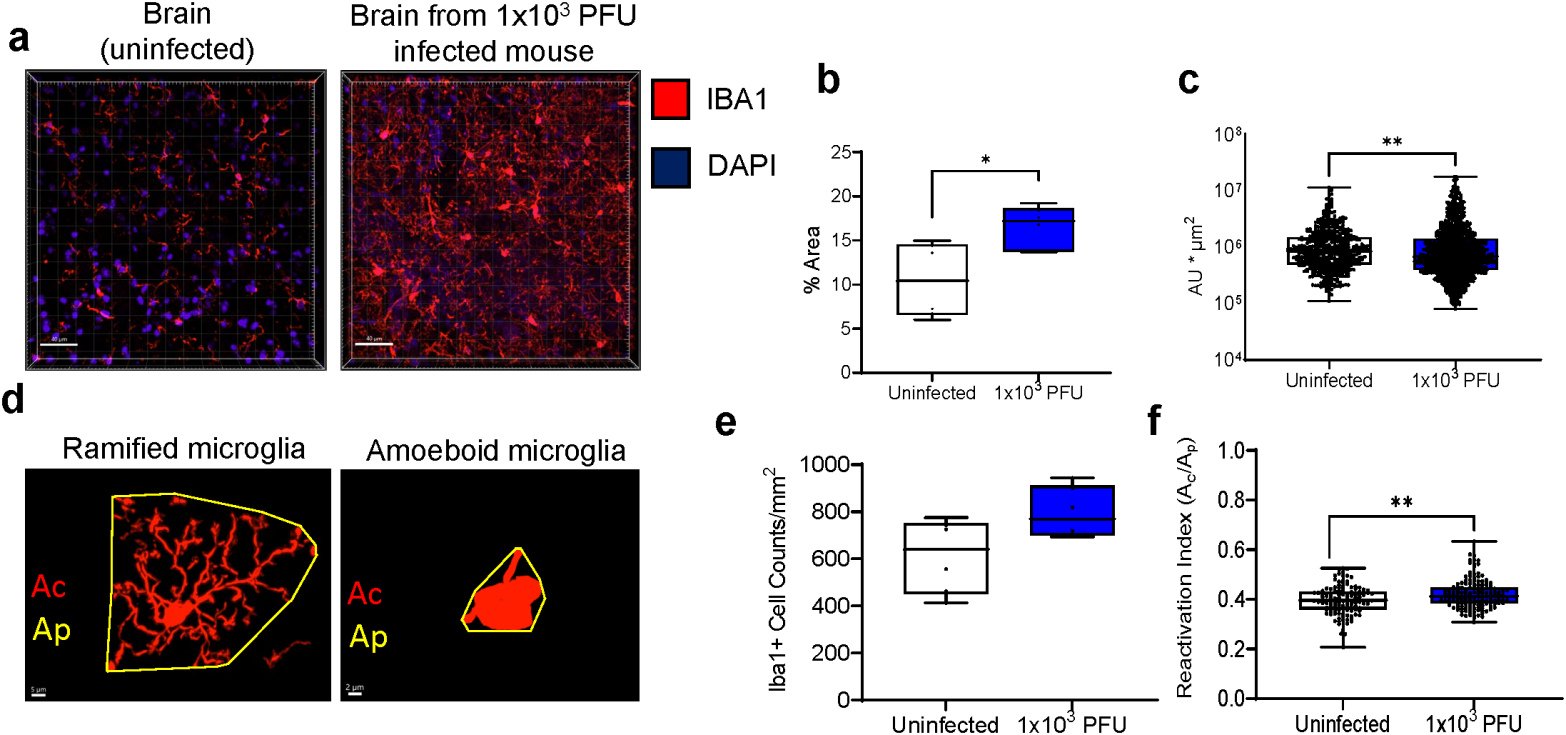
Low inoculum intranasal SARS-CoV-2 infection in mice results in increased pro-inflammatory cytokines in the lung and brain and causes increased microglia reactivity in the brain. **(A)** Confocal microscopy of 30 μm sections of frontal lobe brain parenchyma following low-inoculum infection reveals reactive microglia with increased expression of Iba1 (red) with nuclei stained by DAPI (purple, 3 images/mouse brain at 40Х, n=2/group). **(B)** % area that was Iba1-positive, **(C)** Iba1+ cell counts, **(D)** Examples of microglia morphology Reactive Iba1+ microglia were quantified by **(E)** Iba1 fluorescence intensity, and **(F)** reactivation index between 0 and 1. Ac, cell area; Ap, projection area. Pairwise comparisons by Mann-Whitney U test (*p< 0.05, **p<0.01).

Brains from low inoculum mice showed no detectable viral proteins (checked by spike staining) and no detectable viral transcription (checked using subgenomic E). However, these brains showed reactive microglia, with increased Iba1 expression (as measured by percentage area, fluorescence intensity and reactivation indices, **Figures 5A–F**). Clusters of GFAP+ astrocytes were found in the regions of high Iba1 expression in the brain, suggesting concomitant microgliosis and astrogliosis, as has been reported in human post-mortem samples (**Supplementary Figures 3A, B**). To ask whether parainfectious brain injury with potential blood-brain-barrier damage had taken place, the brain supernatant/serum albumin ratios were measured in uninfected mice and low inoculum infected animals (**Supplementary Figure 3C**). In humans post-COVID-19, NfL has been found to be raised, but there were no significant differences in the brain injury marker NfL, either by ELISA or Simoa (**Supplementary Figure 3D**) at this early time point (day 5 post infection) (7).

### Viral effects on endothelial cells *in vitro* lead to pro-inflammatory cytokine production and subsequent microglia reactivation

**Figure 6:**
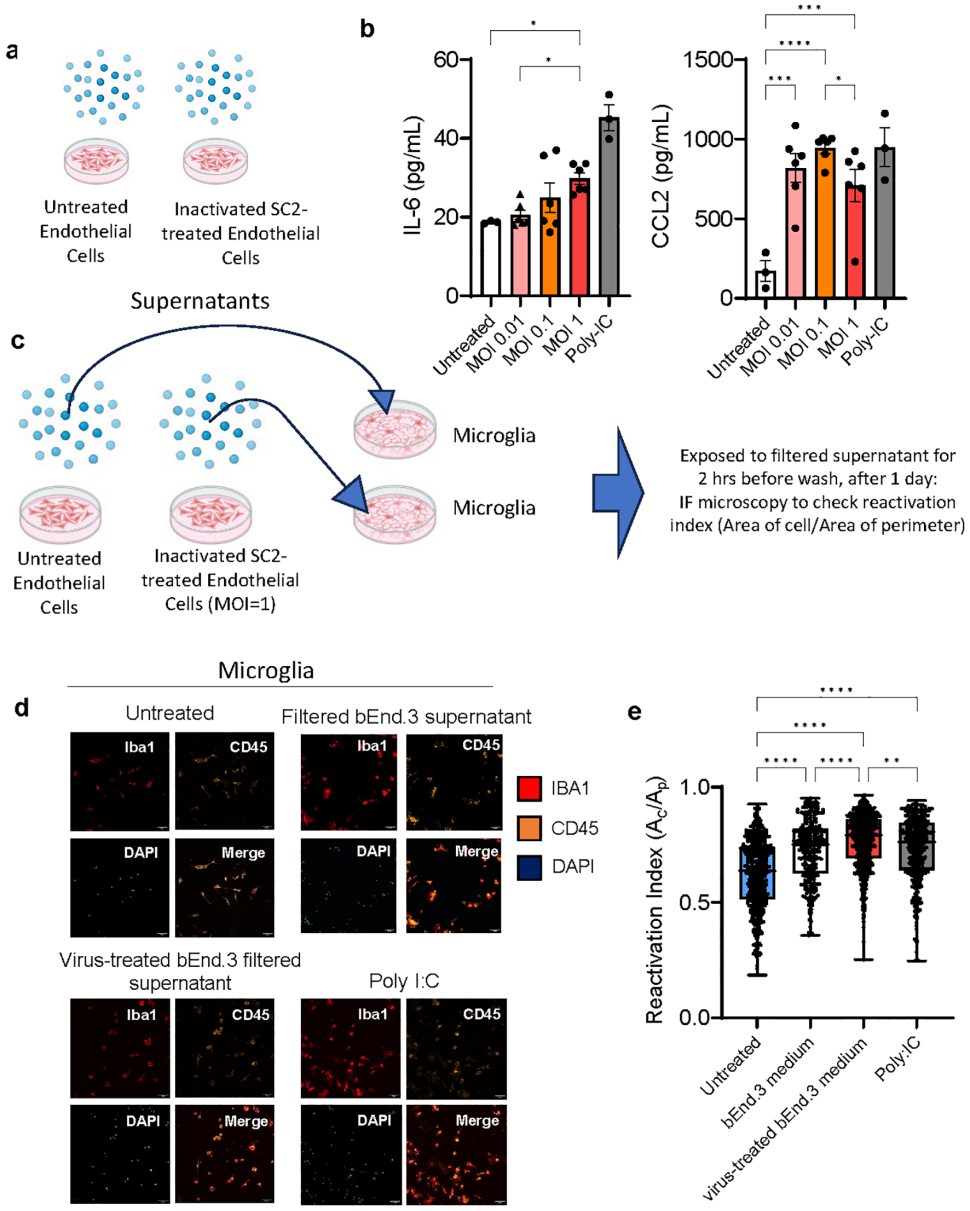
Viral effects on endothelial cells *in vitro* lead to pro-inflammatory cytokine production and subsequent microglia reactivation. **(A, B)** bEnd.3 cells were treated with heat and acid inactivated SARS-CoV-2 at a ratio of 0.01,0.1, or 1 virus copies per cell for 24 hours. At 24 hours concentrations of cytokines IL-6 and CCL2 were determined by ELISA and groups compared by ANOVA *p<0.05, **p<0.01, ***p<0.001, ****p<0.0001. **(C–E)** Primary mouse microglia were treated with 20 nm filtered supernatant taken from bEnd. 3 cells incubated with virus at an MOI of 1, for 2 hours, before being washed and culture medium replaced. At 24 hours cells were fixed and immunostained for Ibal and CD45 and imaged by confocal microscopy. 6-7 separate wells were cultured for each treatment condition and 3-4 for each control condition, with 16 images taken per well at 25x magnification. Dots represent individual cells. Groups compared by Kruskal-Wallis *p<0.05, **p<0.01, ***p<0.001, ****p<0.0001.

We hypothesized that the cerebral vasculature is the most common route for SARS-CoV-2 infection interacting with neuroglial cells and causing local, not necessarily systemic, inflammation. To study the cascade of events in a controlled environment, we exposed mouse endothelial cells (bEnd.3 cell line) to inactivated SARS-CoV-2 viral particles at three multiplicities of infection (MOI), collected and filtered the supernatant, and measured the cytokines present. The TLR3 agonist polyI:C served as a positive control (**Figures 6A, B**). The MOIs of 0.1 and 1 used to treat endothelial cells produced significant amounts of pro-inflammatory cytokines IL-6 and CCL2, so the MOI of 1 was chosen for the subsequent experiments as a way to mirror direct infection of brain endothelial cells, but not microglia and astrocytes (**Figures 6C–E**; **Supplementary Figures 4A, B**). Primary mouse microglia and astrocytes were exposed to the filtered supernatant from endothelial cells and their cytokine production and for microglia, their reactivation index was measured by Iba1+ morphology (**Figures 6D, E**; **Supplementary Figures 4A, B**). The supernatant from MOI=1 exposed endothelial cells resulted in the highest reactivation index indicating a pathway by which SARS-CoV-2 can indirectly affect brain cells and which is consistent with our *in vivo* data.

## Discussion

To better study the acute host response in SARS-CoV-2, and given that SARS-CoV-2 is rarely identified in the brain parenchyma in clinical samples, we developed a low-inoculum mouse model of COVID-19 which induced pulmonary infection in the absence of replication of virus in the brain. Following intranasal administration, by five days the mice had developed evidence of lung infection and immune cell infiltration (18), with production of inflammatory mediators, including CCL2, IL-6, IL-12p40 and IL-1RA; these cytokines were also elevated acutely in the serum of COVID-19 patients with low Glasgow coma scale scores (7). Despite the absence of viral replication in the brain parenchyma, there was some, albeit limited, production of inflammatory mediators in the brain, including CCL4, IFNγ and IL-17A. In addition, there was an increase in microglial Iba1 staining and microglia reactivation morphology. We had expected to also see increased numbers of CD45+ and CD11b+ cells, but believe that this was limited by the thin cryosections examined. In fact, we needed to examine thicker cryosections in order to quantify the microglia morphology (17). In future studies, flow cytometry would be an important way to quantify immune cell infiltration. Immune infiltrates and microgliosis were also observed in our previous mouse studies (19, 20). Parallel *in vitro* studies demonstrated that the filtered supernatant from brain endothelial cells exposed to SARS-CoV-2 virions, induced activation of microglia and production of CCL2. Our *in vitro* model using inactivated virus (due to limitations on CL3 work) delivered viral particles to endothelial cells and we hypothesize that this strongly stimulated them via innate PAMP pathways such as TLRs. Building on this, future studies could apply similar approaches to compare direct and indirect effects of vaccines and active virus on neuroglial cells. Active viruses would stimulate by DAMPs and PAMPs.

Our findings suggest that a primarily pulmonary inflammatory process is rapidly associated with parainfectious immune activation in the brain and the signature of an NK cell and/or T cell response which indicates a cascade of inflammation potentially amenable to treatment. Our mouse model is novel in using a low inoculum of SARS-CoV-2 virus for infection which does not induce lethal brain pathology, allowing us to study the immune activation in the brain in the absence of direct viral invasion. This is congruent with the majority of human autopsy results which show limited virus in the brain, but nevertheless demonstrate inflammation and microglia reactivity (21, 22). Human autopsy studies have studied this concept and found that exposure to lung-derived cytokines is association with microglia activation and that this was reduced by corticosteroid treatment (23). This may also reflect longer term effects, as a hamster model which examined neuropathology at 31 days post-SARS-CoV-2 infection found that Iba1 expression remained elevated (24). Studies of brain organoids have reported that Iba1+ microglia engulf post-synaptic material contributing to synapse elimination (25).

There have been reports of viral encephalitis and neuron degeneration and apoptosis observed in non-human primates (26, 27). Interestingly, in these studies the virus was present at low amounts in the brain and was found predominantly in the vasculature as visualized by co-localization with Von Willebrand Factor (27). Mimicking the clinical scenario, there was no correlation found between neurological markers with severity of respiratory disease. Another study reported increased CCL11 (eotaxin) in mouse serum and CSF that correlated with demyelination (28). That mouse model also lacked direct viral neural invasion by infecting mice that were intratracheally transfected with human ACE2. The demyelination was also observed after intraperitoneal administration of CCL11. Interestingly, clinical studies showed higher plasma levels of CCL11 in the patients who had brain fog (28). We did not observe elevated serum cytokines in our studies and this could be due to severity, timepoint, and/or technical differences. Our negative results in the serum are at least in part contributed to by heat-inactivation of experimental samples, which were part of safety protocols in the CL3 lab at the point when these experiments were conducted (29). Hamster studies have showed that COVID-19 leads to IL-1β and IL-6 expression within the hippocampus and medulla oblongata and is associated with decreased neurogenesis in the hippocampal dentate gyrus which leads to learning and memory deficits (30). This has also been shown in direct *in vitro* assays—with application of serum from COVID-19 patients with delirium with elevated IL-6 leading to decreased proliferation and increased apoptosis of a human hippocampal progenitor cell line (31). Our *in vitro* studies enabled us to isolate a potential mechanism by which SARS-CoV-2 indirectly affects brains cells—by studying endothelial cells which express ACE2 and can be directly infected by the virus (15), collecting their supernatants containing pro-inflammatory cytokines, and exposing microglia and astrocytes to them. Spike protein alone has previously been found to cause inflammation and associated cognitive deficits in animal models which is a common and important long-lasting symptom of COVID-19 in humans (32–34).

In conclusion, the low inoculum SARS-CoV-2 mouse model and parallel *in vitro* studies highlight an approach to study parainfectious effects on the brain and enables characterisation of the neuroglial cells themselves. The cytokine signature and microglia reactivity post infection indicate an acute local immune response including initial inflammation in the absence of active viral replication in the brain that could be amenable to targeted immunosuppression which can direct future studies.

## Data Availability

The raw data supporting the conclusions of this article will be made available by the authors, without undue reservation.
