# Supplementary figures and images for: Pulmonary SARS-CoV-2 infection leads to para-infectious immune activation in the brain
Source: Front Immunol. 2024 Oct 14;15:1440324. doi: 10.3389/fimmu.2024.1440324 (PMC11519853; doi:10.3389/fimmu.2024.1440324)

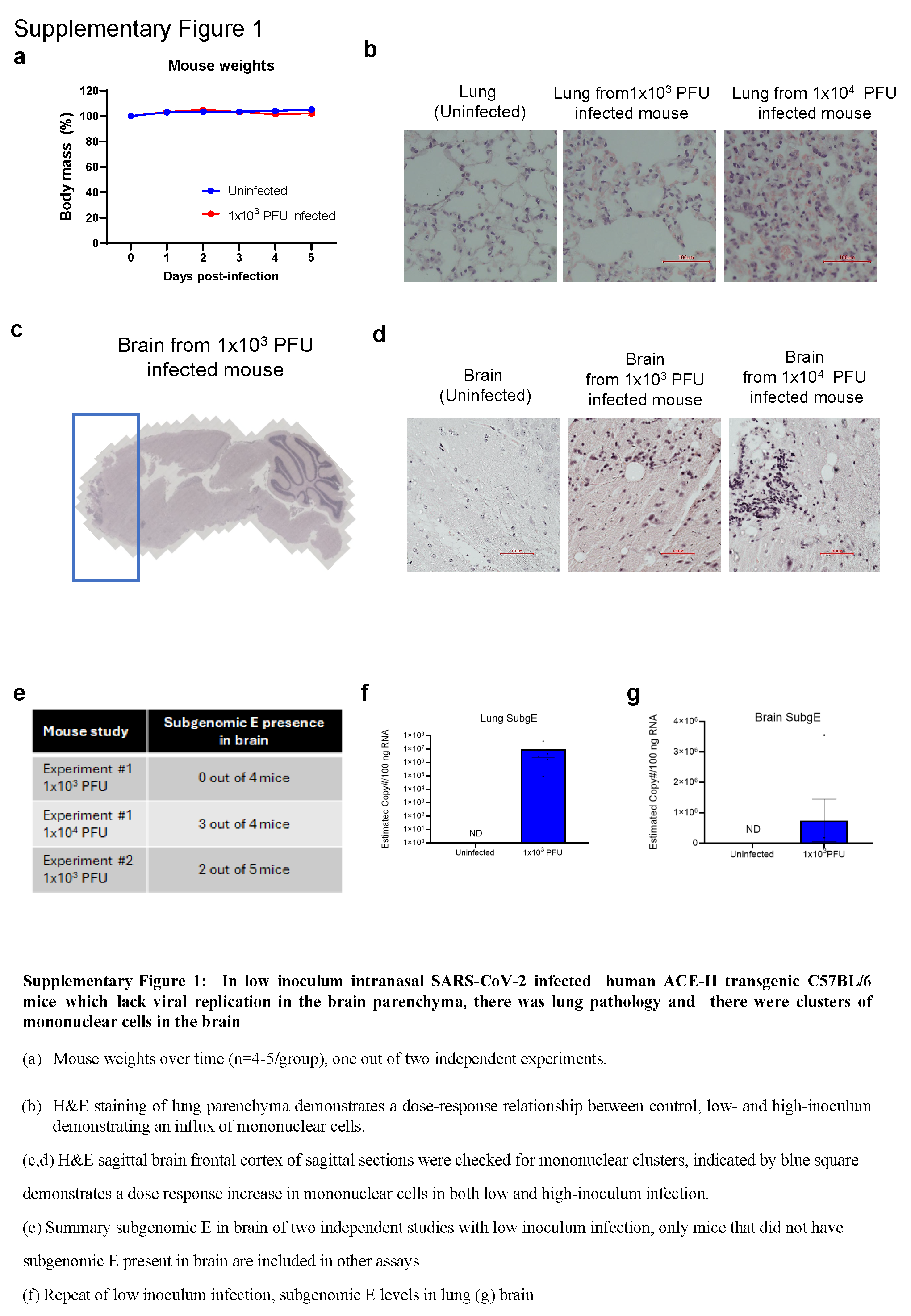

Supplement: Supplementary file 1 [file Image1.tiff]

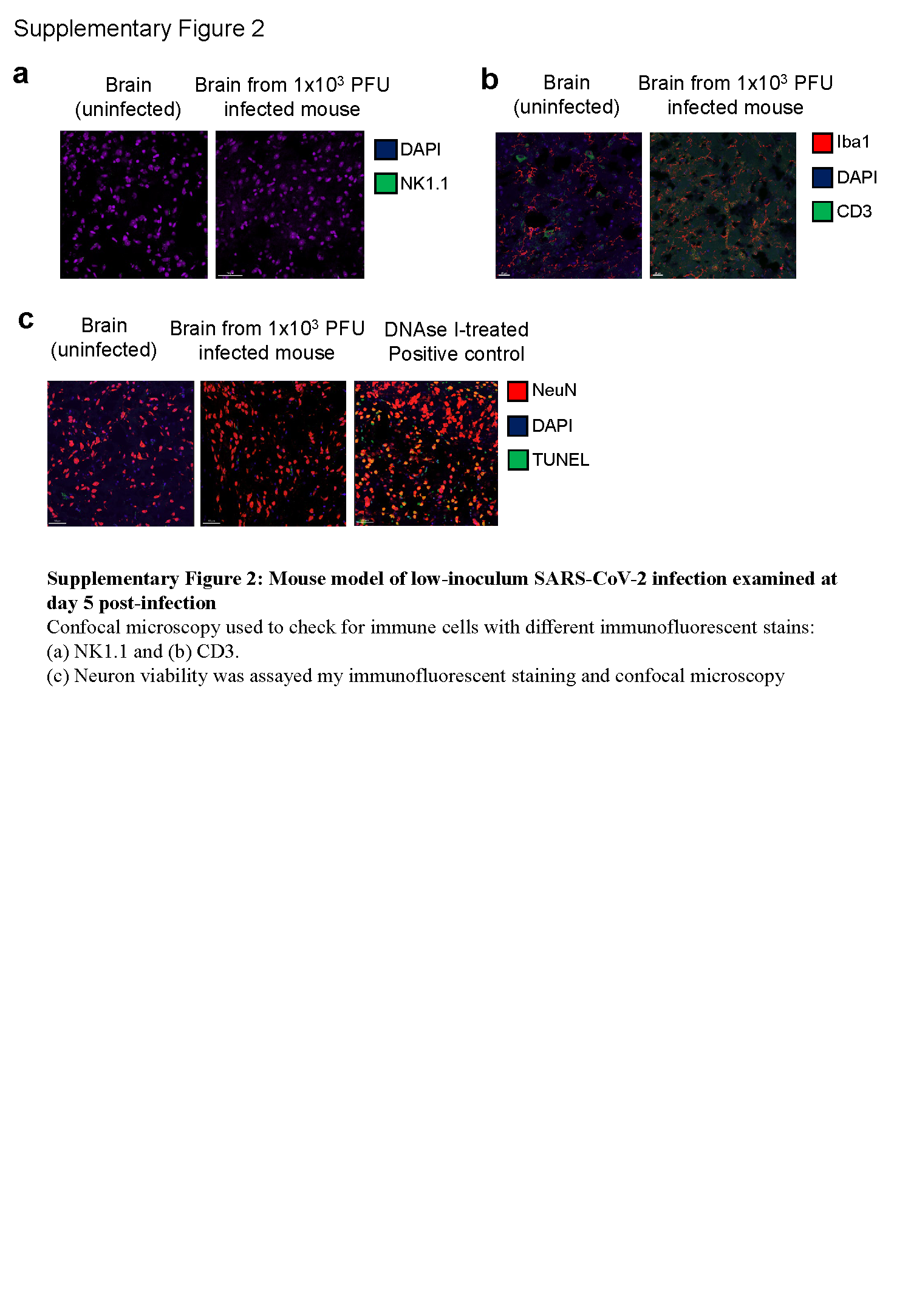

Supplement: Supplementary file 2 [file Image2.tiff]

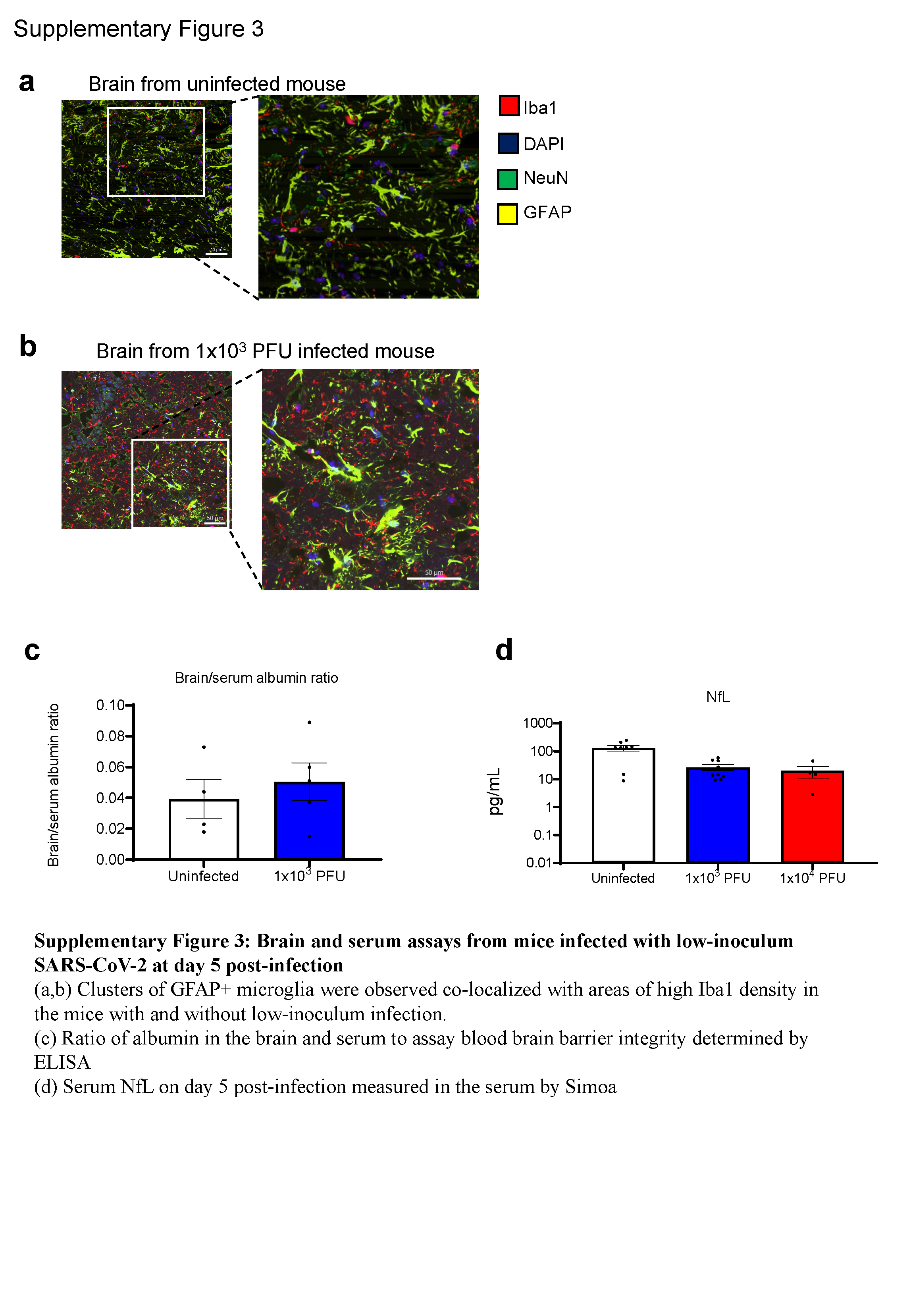

Supplement: Supplementary file 3 [file Image3.tiff]

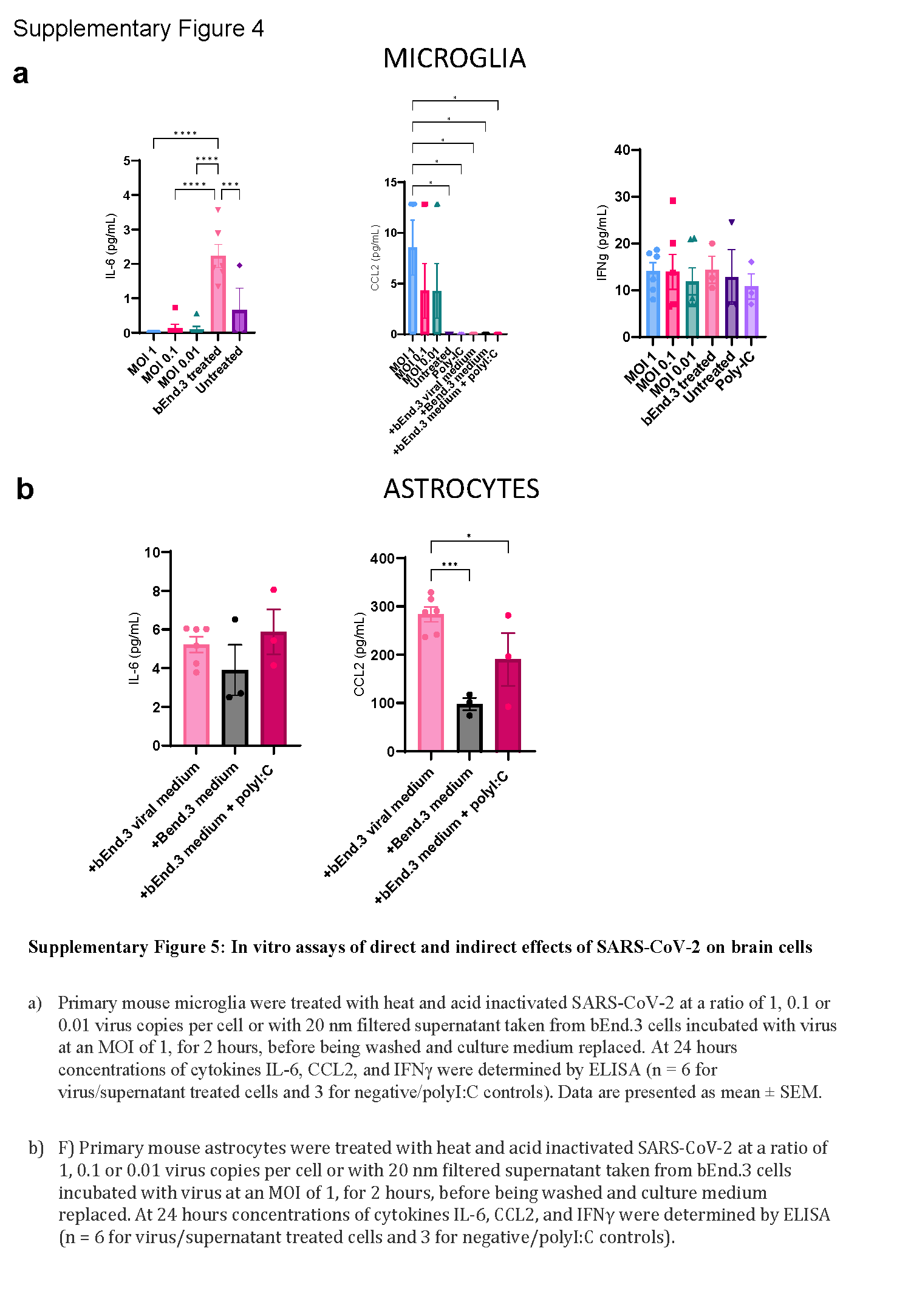

Supplement: Supplementary file 4 [file Image4.tiff]

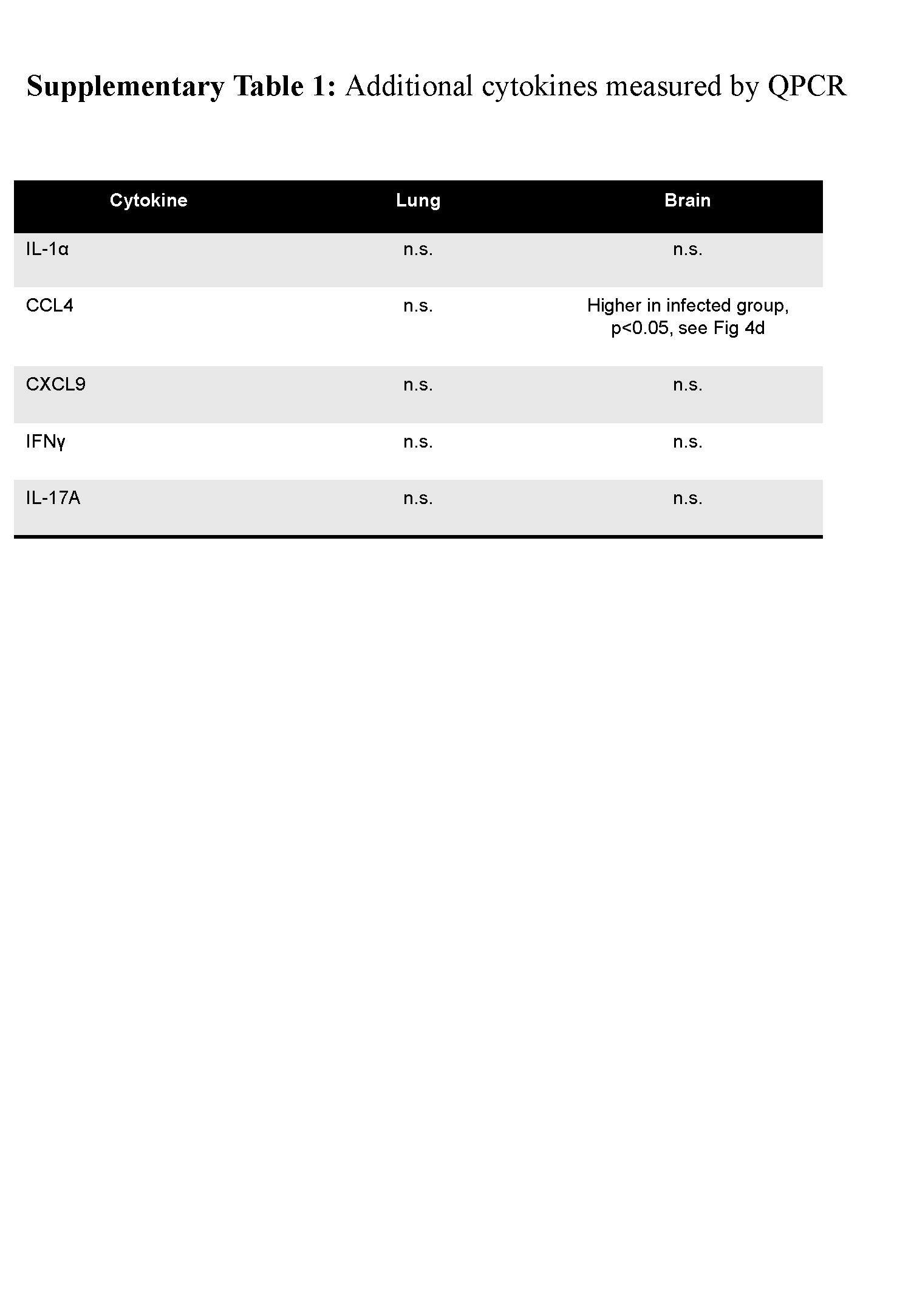

Supplement: Supplementary file 5 [file Image5.tiff]

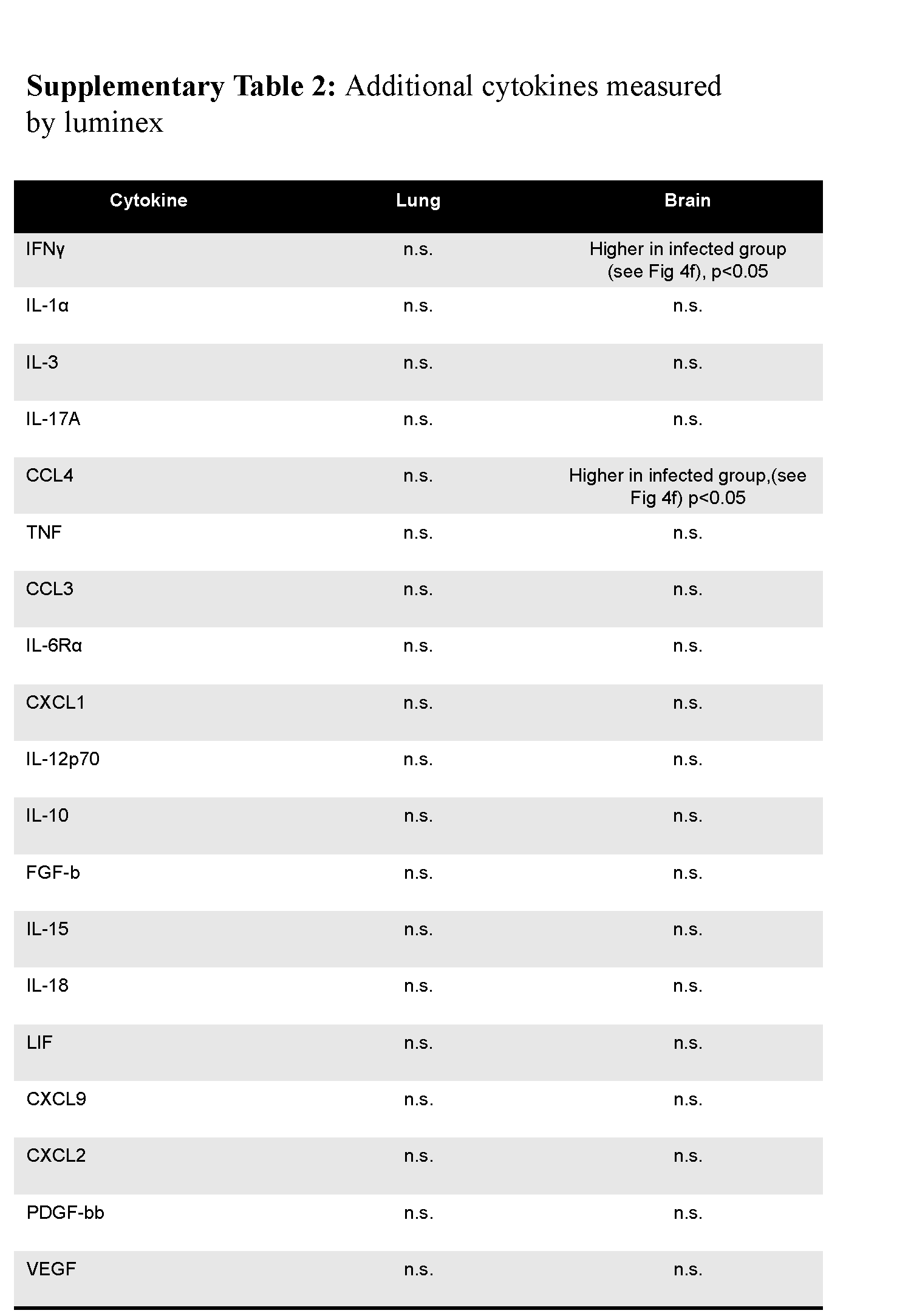

Supplement: Supplementary file 6 [file Image6.tiff]
